# Bioinformatic Analysis Reveals Central Role for Tumor-Infiltrating Immune Cells in Uveal Melanoma Progression

**DOI:** 10.1155/2021/9920234

**Published:** 2021-06-11

**Authors:** Mieszko Lachota, Anton Lennikov, Karl-Johan Malmberg, Radoslaw Zagozdzon

**Affiliations:** ^1^Department of Clinical Immunology, Doctoral School, Medical University of Warsaw, 02-006 Warsaw, Poland; ^2^Department of Ophthalmology, Schepens Eye Research Institute of Massachusetts Eye and Ear, Harvard Medical School, Boston, Massachusetts, USA; ^3^Department of Cancer Immunology, Institute for Cancer Research, Oslo University Hospital, 0310 Oslo, Norway; ^4^Center for Infectious Medicine, Department of Medicine Huddinge, Karolinska Institutet, Karolinska University Hospital, 141 86 Stockholm, Sweden; ^5^Department of Clinical Immunology, Medical University of Warsaw, 02-006 Warsaw, Poland; ^6^Department of Regenerative Medicine, The Maria Sklodowska-Curie National Research Institute of Oncology, 02-781 Warsaw, Poland

## Abstract

Tumor-infiltrating immune cells are capable of effective cancer surveillance, and their abundance is linked to better prognosis in numerous tumor types. However, in uveal melanoma (UM), extensive immune infiltrate is associated with poor survival. This study aims to decipher the role of different tumor-infiltrating cell subsets in UM in order to identify potential targets for future immunotherapeutic treatment. We have chosen the TCGA-UVM cohort as a training dataset and GSE22138 as a testing dataset by mining publicly available databases. The abundance of 22 immune cell types was estimated using CIBERSORTx. Then, to determine the significance of tumor-infiltrating cell subsets in UM, we built a multicell type prognostic signature, which was validated in the testing cohort. The created signature was built upon the negative prognostic role of CD8+ T cells and M0 macrophages and the positive role of neutrophils. Based on the created signature score, we divided the patients into low- and high-risk groups. Kaplan-Meier, Cox, and ROC analyses demonstrated superior performance of our risk score compared to either clinical or pathologic characteristics of both cohorts. Further, we found the molecular pathways associated with cancer immunoevasion and metastasis to be enriched in the high-risk group, explaining both the lack of adequate immune surveillance despite increased infiltration of CD8+ T cells as well as the higher metastatic potential. Genes associated with tryptophan metabolism (IDO1 and KYNU) and metalloproteinases were among the most differentially expressed between the high- and low-risk groups. Our correlation analyses interpreted in context of published in vitro data strongly suggest the central role of CD8+ T cells in shifting the UM tumor microenvironment towards suppressive and metastasis-promoting. Therefore, we propose further investigations of IDO1 and metalloproteinases as novel targets for immunotherapy in lymphocyte-rich metastatic UM patients.

## 1. Introduction

Uveal melanoma (UM) is the most common primary eye cancer in adults and the second most common type of melanoma. The mainstay of current treatment based on either enucleation or brachytherapy can achieve local disease control in most patients. However, despite excellent local management, ~50% of the patients still develop distant metastases, with the liver being the most common site [1–4]. Current treatment regimens are ineffective in increasing overall survival in metastatic UM with a median of 12 months, creating an urgent demand for novel and effective therapies [2, 3, 5]. Strikingly, for the last 40 years, the survival times did not improve, reflecting a lack of progress in developing novel treatment strategies [3, 4].

Infiltration of immune cells is linked with improved prognosis in numerous different cancer types [6, 7]. Immune cells present in the tumor milieu can suppress tumor growth and induce disease regression, primarily through cell-mediated cytotoxicity [6, 8, 9]. CD8+ T cells and natural killer (NK) cells are primary effector cells in antitumor response, capable of inducing cancer cell death and mounting a robust immune response against the tumor [6, 10]. However, in the course of cancer microevolution, neoplastic cells undergo a series of adjustments adapting the cells to increased proliferation and selective pressure from the immune system [11, 12]. These changes induce the formation of a hostile tumor microenvironment (TME) capable of inhibiting the immune cell effector function [11–13]. Hypoxia, immunosuppressive cytokines, metabolites, and other factors present in the TME can effectively dampen antitumor response, allowing for unrestricted tumor growth [12–16]. Therefore, novel strategies emerging in cancer research are aimed at targeting the TME in order to restore proper immunosurveillance. Checkpoint inhibition representing such an approach has been proven effective in the treatment of several cancers, achieving spectacular success in the therapy of cutaneous melanoma (CM) [17].

Even though both uveal and cutaneous melanoma arise from melanocytes, they have distinct clinical and biological characteristics due to differences in the transformation process, oncogenic drivers, and anatomical location [18–20]. While CM exhibits the highest somatic mutation rate across all the cancers, UM is characterized by a low mutational burden, partially explaining the discrepancy between the outcomes of checkpoint inhibitor-based therapies [18–24]. Other targeted treatments, such as antiangiogenic therapies, have also failed to provide substantial antitumor effects in clinical trials [24–26]. Moreover, the prognostic role of immune infiltrating cells in UM appears to be different compared to CM. Paradoxically, increased abundancy of tumor-infiltrating cells, particularly T cells, is associated with poor prognosis and increased metastatic potential in UM [8, 27–29].

UM has the benefit of growing in the immune privilege site. The eye is protected from the immune system's overactivation by numerous immunosuppressive mechanisms such as the blood-eye barrier limiting the influx of immune cells and other mechanisms including high expression of TGF-*β* and IDO1 in aqueous humor [14, 30–32]. While beneficial in physiological settings, these characteristics are unfavorable in intraocular neoplastic disease due to suppressing the antitumor immune response and potentiating tumor growth [30, 32].

The negative prognostic role of immune infiltrating cells in UM, first discovered nearly 30 years ago, remains an unexplained phenomenon. We perform comprehensive mining of the TCGA and GEO database to determine the role of different immune cell subsets in the prognosis of UM patients. By employing the primal-dual active set-based covariate selection algorithm “BeSS,” we create a 3-cell type model associated with poor prognosis. Further, we compare the composition of tumor-infiltrating cells between high- and low-risk groups, evaluate differentially expressed genes (DEGs) and enriched pathways, and integrate our data with the current state of knowledge to delineate the molecular mechanisms of UM immunosuppressive milieu as well as immune cells' protumorigenic activity in UM.

## 2. Materials and Methods

### 2.1. Data Mining

The gene expression profiles of UM from 80 patients, together with their clinical and survival data, were downloaded from TCGA Xena Hub (https://tcga.xenahubs.net) with cohort name: GDC TCGA-UVM for htseq-counts type and TCGA-PANCAN filtered for UVM/SKCM patients for harmonized TOIL RSEM TPM type. The chromosome aberration status in TCGA-UVM cohort was derived from the original TCGA-UVM manuscript [33]. Further, we searched the GEO database by looking for studies with (1) available gene expression profiling data and (2) available survival data [34]. We chose a dataset with the most cases, resulting in GSE22138 with 63 patients being used in this study [35]. GSE22138 data was downloaded using “GEOquery” [36, 37]. TCGA-UVM was used as the training cohort, and the GSE22138 was reserved for testing the created model. TCGA-PANCAN TOIL RSEM TPM data filtered for patients from the SKCM cohort was used as a comparison against UM patients [38]. The harmonized TCGA-PANCAN data filtered by cancer type was used for deconvolution to enable direct comparisons between cancer types. “biomaRt” was used to annotate Ensembl/Affymetrix probe IDs with HGNC gene symbols [39]. Samples with a censoring time of fewer than 60 days were excluded to avoid introducing mixed factors.

### 2.2. Estimation of Immune Cell Type Abundance in Tumor Tissue

We used CIBERSORTx (https://cibersortx.stanford.edu/) to evaluate the relative abundance of predefined cell types in mixed solid tissues [40]. Normalized and harmonized RNA-seq TOIL RSEM TPM gene expression data from tumor tissues in the TCGA-PANCAN cohort was filtered by UVM/SKCM sample IDs and used for this analysis [41]. We employed the LM22 leukocyte gene signature matrix downloaded from the CIBERSORTx website. LM22 contains 547 genes distinguishing 22 types of immune-related cells. As recommended, we disabled quantile normalization for deconvolution of RNA-seq data in the training and cutaneous melanoma cohort and enabled for microarray data in the testing cohort. We set the number of permutations to 1000 for robust analysis. The B-mode of batch correction supplied with LM22 GEP was used. CIBERSORTx enumerated the abundance scores of the 22 infiltrating immune cells, including B cells, dendritic cells, T cells, natural killer cells, macrophages, and others. To simplify the text, we referred to the cell type abundance estimates as “abundance” or “infiltration” alone. The results were filtered with a *p* value < 0.05 threshold. The Wilcoxon rank-sum test was applied to evaluate the differences in cell proportions between high- and low-risk groups.

### 2.3. Creation and Validation of Prognostic Immune Cell Signature

We used “BeSS” R package utilizing the primal-dual active set-based approach to select variables for the multicell type model [42]. Significantly, to minimize the risk of model overfitting, the maximum number of predictors was limited to 3 variables. All of the cell types were included in the screening with CD8+ T cells being forced in the model due to their established negative prognostic role and crucial function in tumor immunology. In addition to CD8+ T cells, M0 macrophages and neutrophils were returned as best candidates, and after evaluation in multivariate Cox analysis, they were used in establishing the final model. Each patient's risk score was calculated by a sum of the abundance estimate of each immune cell type weighted by its multivariate Cox regression coefficient. Using the median risk score as the cutoff point, the patients in the training cohort were distributed to either high- or low-risk group. Kaplan–Meier analysis and log-rank test were performed to evaluate the survival difference between the two groups. Cox and ROC analyses were further used to assess the immune cell type signature's prognostic value in the training cohort. The immune cell type signature score in the testing and cutaneous melanoma cohort was calculated using the same formula as in the training cohort.

### 2.4. Gene Expression Data Processing

The RNA-seq expression data was HTSeq-counts type. After assigning the samples to two groups based on risk score, we performed differential gene expression analysis using “DESeq2” in R [43]. To optimize power, we used Independent Hypothesis Weighting (IHW) with an adjusted *p* value threshold < 0.05 for reporting DEGs and for further gene-set enrichment analysis [44].

### 2.5. Gene Set Enrichment Analysis

Active subnetwork search and enrichment analysis were done using the pathfindR package in R, accordingly to the authors' recommendations [45]. MSigDB Hallmark (*H*) set was to obtain gene sets for enrichment analysis [46]. DEGs obtained from DESeq2 with additional IHW were filtered for statistical significance using adjusted *p* value < 0.05 as a threshold and used as an input [44].

### 2.6. Statistical Analysis

Statistical calculations in this study were performed using R [47]. “tidyverse” was used in preparations of all the figures [48]. Kaplan–Meier analysis was used to examine the prognostic differences between the high- and low-risk groups. The *p* value was assessed by the two-sided log-rank test. Univariate Cox analysis was conducted to illustrate the relationship between the immune cell type signature risk score and UM prognosis. “survival” R package was used in survival analyses [49]. The ROC curves were calculated and plotted by the “pROC” package to evaluate the risk score's sensitivity and specificity for prognosis prediction [50]. The area under the curve (AUC) was used as a measurement of prognostic accuracy. Normality was assessed using Shapiro-Wilk test. Because the expression of genes of interested did not follow normal distribution, we used Spearman's rank correlation to assess the relationship between gene expression. The Wilcoxon rank-sum test was applied to evaluate the differences in cell proportions between high- and low-risk groups. In all analyses, *p* < 0.05 was considered a statistically significant threshold.

### 2.7. Visualization

The flowchart was created in Lucidchart (http://www.lucidchart.com/). The Kaplan-Meier plots were created using “survmeier” R package and the ROC plots with “pROC” R package [50, 51]. Correlation plots were created with “ggpubr” and “corrplot” R packages [52, 53]. The heat map was created with “ComplexHeatmap” R package [54]. The rest of the figures were created with “ggplot2” [48].

## 3. Results

### 3.1. Cohort Characteristics

**Figure 1:**
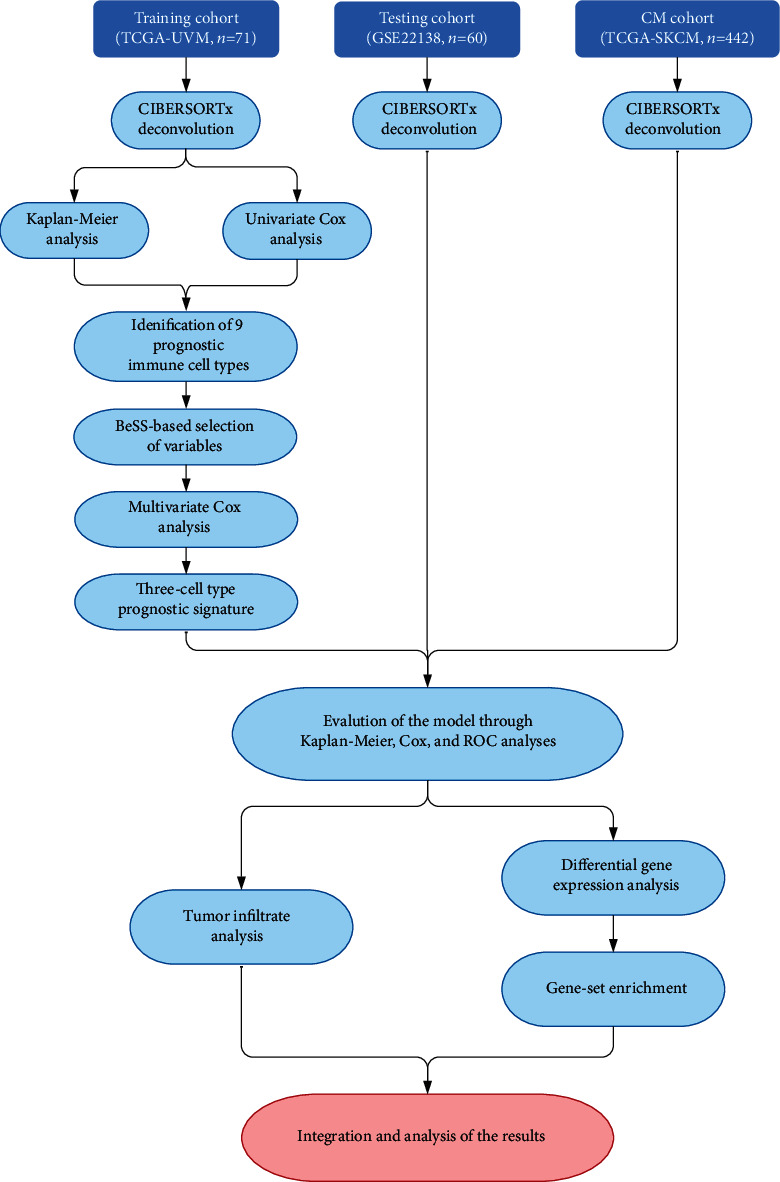
A simplified flowchart of the study.

A simplified flowchart of the study is presented in Figure 1. After filtering by censor time and CIBERSORTx deconvolution's *p* value, 71 cases from the TCGA-UVM dataset were used as a training dataset, 60 from GSE22138 as a testing dataset, and 442 patients from the CM TCGA-SKCM dataset were used as a comparison to TCGA-UVM. Chosen clinical and pathological characteristics of training and testing cohorts are summarized in Table 1.

### 3.2. Estimation of the Intratumoral Abundance of 22 Immune Cell Subtypes

To enumerate the tumor-infiltrating immune cells, we utilized the CIBERSORTx algorithm, which through *ν*-support vector regression (*ν*-SVR) can accurately estimate cell abundance based on the supplied gene expression profile [40]. We used the previously validated LM22 gene signature, consisting of 22 cell types and 547 genes to analyze the proportions of tumor-infiltrating immune cells in all cohorts. A visual summary of subset proportions in the training cohort is presented in Supplementary Figure 1A. Additionally, we calculated the correlation coefficients between all of the cell subsets, which were taken into considerationduring model design (Supplementary Figure 1B).

### 3.3. Prognostic Impact of Immune Cell Types in Uveal Melanoma

**Figure 2:**
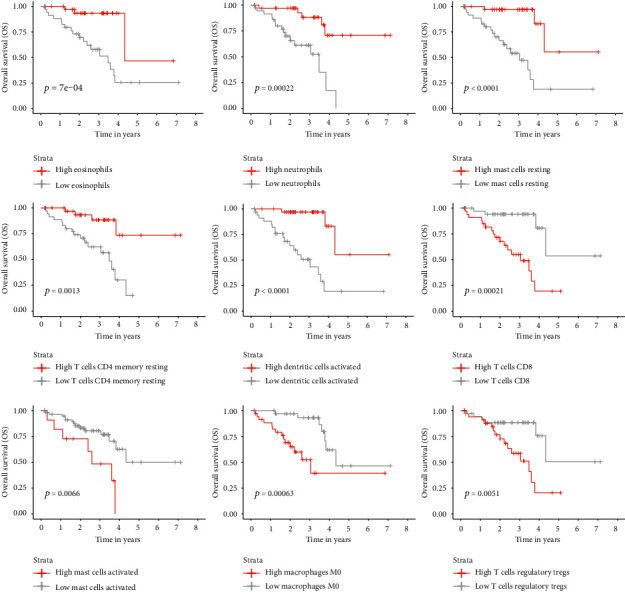
Kaplan-Meier survival analysis for training cohort based on median scores for each immune cell type. Only cell types characterized by a statistically significant prognostic effect (*p* < 0.05) are displayed.

Kaplan-Meier and univariate Cox regression analysis were performed on the training cohort to assess the significance of different immune cell types in UM as overall survival (OS) predictors. Patients were split into two groups based on the median abundance of each cell type. Nine immune cell types were identified in a Kaplan-Meier analysis as a significant determined by *p* value calculated with log − rank test < 0.05. Cell types with positive prognostic value included neutrophils, eosinophils, resting mast cells T CD4+ memory resting, and activated dendritic cells, whereas CD8+ T cells, regulatory T cells (Tregs), M0 macrophages, and activated mast cells were identified as negative prognostic factors (Figure 2).

These findings were supported by Cox univariate analysis where CD8+ T cells were characterized by the highest hazard ratio (HR = 6.24, 95% CI = 2.08‐18.75, *p* = 0.0011), whereas resting Mast cell signature was the strongest factor associated with improved survival (HR = 0.11, 95% CI = 0.03‐0.36, *p* = 0.0003) (Supplementary Table 1). The unexpected negative prognostic role of CD8+ T cells, which are the main effector cells in anticancer immune response, prompted our further investigations.

### 3.4. Construction and Assessment of Prognostic Model Build Based on Immune Cell Types

We utilized a primal-dual active set-based approach implemented in the BeSS R package to select cell types for the prognostic model. Due to established biological and prognostic significance, CD8+ T cells were forced in the model as described in detail in Materials and Methods. The three best variable candidates returned by BeSS were further evaluated in a multivariate Cox regression analysis. The final cell types included in the model were CD8+ T cells, M0 macrophages, and neutrophils. Each patient's risk score was created by a sum of included immune cell abundance estimates weighted by their coefficients. Then, based on median score, the patients were assigned either to high- or low-risk group.

**Figure 3:**
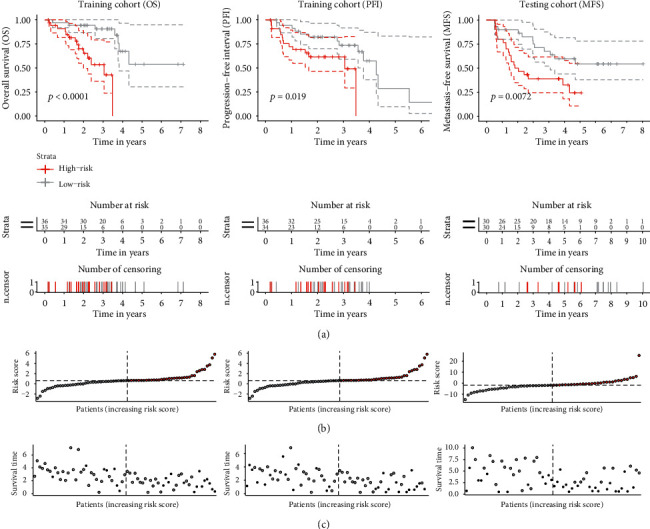
(a) Kaplan–Meier survival analysis based on the 3-cell type signature in training cohort (overall survival and progression-free interval), and testing cohort (metastasis-free survival). Differences between the groups were detected by log-rank test. (b) Distribution of immune cell-based score in both cohorts. (c) Survival time in a function of risk score, stratified by event occurrence (black) in training cohort (overall survival and progression-free interval), and testing cohort (metastasis-free survival).

Kaplan-Meier survival analysis showed that the patients' overall survival in the high-risk group was significantly shorter than in low-risk groups (*p* < 0.0001) (Figure 3(a)). The unfavorable effect of the high-risk immune signature was also observed in progression-free interval (PFI) in the training cohort (*p* = 0.019). To validate the prognostic accuracy of the created immune score, we evaluated the immune cell score in the testing cohort using the same method for score calculation and group assignment. In agreement with previous results, the high-risk group showed significantly decreased metastasis-free survival (MFS) time (*p* = 0.0072) (Figure 3(a)). Unfortunately, the OS data was not available in the testing dataset, whereas MFS data was not available in the training cohort, making a direct comparison between the training and testing cohort impossible. We found no other publicly available UM transcriptomic datasets with OS data and more than 30 patients. Nevertheless, development of metastasis is the most critical event in the course of UM [1, 3]. While metastatic disease is not equivalent to death, almost no disease-specific death occurs without distant metastases, implicating a firm relationship between the two outcomes in UM [55].

The distribution of risk scores and a survival time in a function of risk score stratified by event occurrence (OS) are shown in Figures 3(b) and 3(c) for both training and testing cohort, respectively. We observed shorter survival time and an increased number of events in the high-risk group in both cohorts.

**Figure 4:**
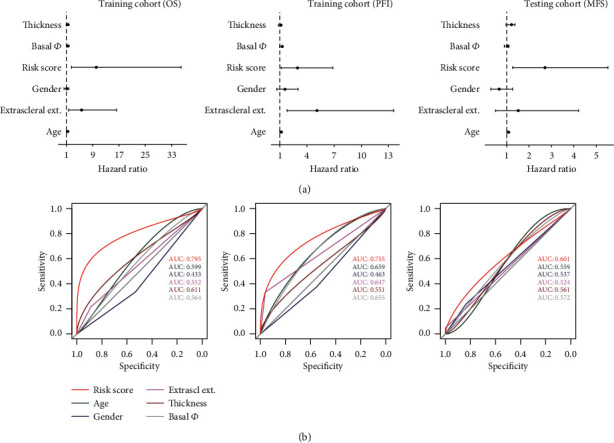
Forest plot summary of univariate Cox analysis of created score's prognostic value (a) and receiver operating characteristic (ROC) analysis of the sensitivity and specificity of the prognosis prediction score (b) in training cohort (overall survival), training cohort based (progression-free interval), and testing cohort based (metastasis-free survival). Additional clinical and pathologic parameters such as age, gender, tumor thickness, basal tumor diameter, and extrascleral extension were included as a comparison.

For an additional assessment of the model, we used receiver operating characteristic (ROC) analysis and univariate Cox regression to compare the created score against multiple clinical and pathologic features, including patient's age at diagnosis, tumor diameter, tumor thickness, extrascleral extension of the tumor, and patient's gender. In the training cohort, Cox analysis showed superior performance of created score compared to other variables based on strong association with OS (HR = 9.66731, 95% CI = 2.60306‐35.90275, *p* = 0.0007) and PFI (HR = 2.7943, 95% CI = 1.1399‐6.84985, *p* = 0.02469). Consistent with these results, immune cell score displayed a close association with MFS in testing cohort (HR = 2.64739, 95% CI = 1.26927‐5.52181, *p* = 0.00944) (Figure 4(a)). Further, we performed ROC analysis with a 3-year cutoff as an assessment of the immune cell score's prognostic value. The area under the curve (AUC) in the training cohort was 0.795 for OS and 0.755 for PFI, which was superior to all the other parameters (Figure 4(b)). In the ROC analysis in the testing cohort, the score exhibited moderate performance with AUC = 0.601.

Nevertheless, it remained superior to other clinical and pathologic parameters (Figure 4(b)). We speculate that the decrease in prognostic value may be partially due to different outcomes measured (OS/PFI vs. MFS) as well as the technical differences between the data (RNA-seq vs. microarray).

Additionally, we assessed the correlation between created risk score and commonly detected chromosomal aberrations with significant prognostic value in UM - 8q segment gain and chromosome 3 loss. We observed a modest correlation between the risk score and both abnormalities (Supplementary Figure 3). Moreover, we divided the training cohort into subgroups based on 8q segment and chromosome 3 status to reassess our model's performance depending on the genomic aberrations. In both subgroups associated with poor prognosis, chromosome 3 monosomy, and 8q segment gain, the model performed similarly as in the whole cohort. At the same time, it lacked prognostic value in tumors without the high-risk chromosomal abnormalities (Supplementary Table 2).

### 3.5. Comparison of the Prognostic Value of Immune Cells between Uveal and Cutaneous Melanoma

**Figure 5:**
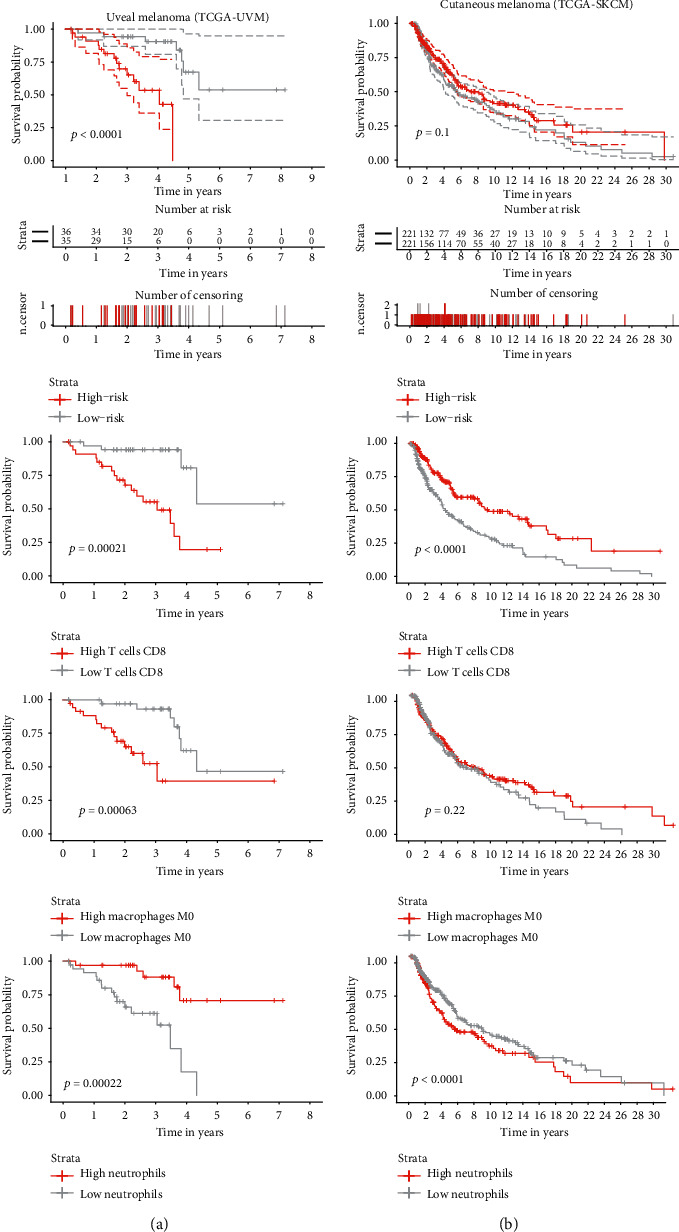
Comparison of Kaplan–Meier overall survival (OS) analysis based on the 3-cell type signature and each individual cell type separately between uveal (a) and cutaneous (b) melanoma. Differences between the groups were detected by log-rank test.

To get a broader perspective of UM's immunobiology, we have decided to assess our immune cell signature's performance and its individual components (CD8+ T cells, M0 macrophages, neutrophils) on the CM cohort. Intriguingly, although not statistically significant, the score tended to associate with improved survival, and the trend was reversed. The prognostic impact of all three cell types included in the generation of the score was reversed, with the effects of CD8+ T cells and neutrophils being statistically significant (*p* < 0.0001 and *p* = 0.048, respectively) (Figure 5).

### 3.6. The Immune Architecture of the Tumors in High- and Low-Risk Group

**Figure 6:**
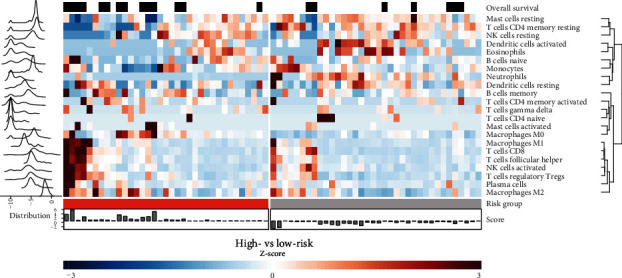
Distribution of immune cells across the samples in training cohort, stratified by assignment to high- (red) and low-risk (gray) groups. Additionally, histogram displaying the distribution of estimated immune cell abundance is plotted on the left (log-transformed), deviation of prognostic score's value from the median on the bottom, and occurrence of the event (black) on the top.

Comprehensive visualization of the TME's cellular architecture is presented in Figure 6. Immunosuppressive cells such as Tregs were enriched in the high-risk group, while resting CD4+ memory T cells and activated dendritic cells dominated in the low-risk patients (Figure 6 and Supplementary Figure 2).

**Figure 7:**
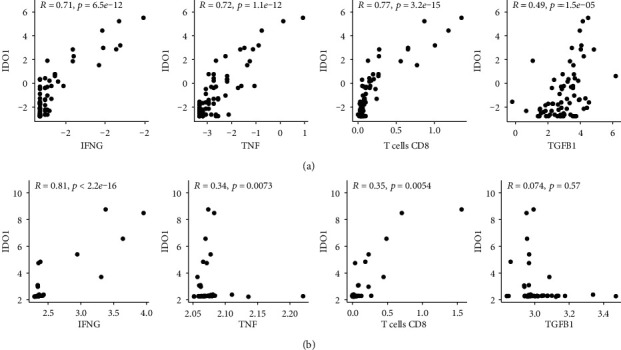
Correlation between IDO1 expression and IFNG, TNF, CD8+ T cell score and TGFB1 (left to right) in training cohort (a) and testing cohort (b). Displayed values are log transformed (log2(*x* + 0.1)).

**Figure 8:**
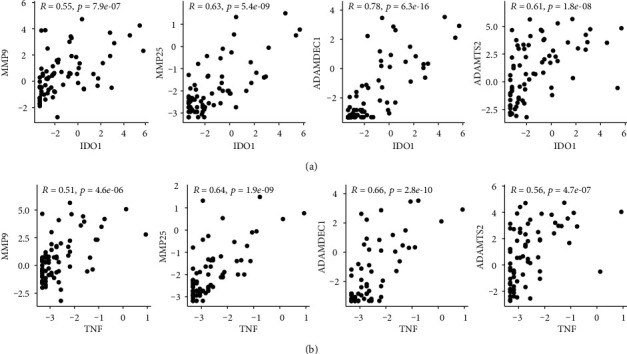
Correlation between IDO1 (a) or TNF (b) expression with MMP9, MMP25, ADAMDEC1, and ADAMTS2 (left to right) in the training cohort. Displayed values are log transformed (log2(*x* + 0.1)).

In order to investigate the underlying immunological differences between the high- and low-risk groups that could explain the unusual negative prognostic value of the immune cell type signature, we performed differential gene expression analysis with DESeq2. After independent hypothesis weighting (IHW) and filtering with an adjusted *p* value threshold < 0.05, we obtained 722 DEGs. One of the top DEGs was metalloproteinases and genes associated with tryptophan metabolism (IDO1, KYNU). Importantly, all of the statically significant DEGs encoding metalloproteinases were upregulated in the high-risk group (Table 2). To investigate the reasons behind increased IDO1 expression in the high-risk group, we have calculated the correlation between IDO1 and IFN-*γ* (IFNG), TNF-*α* (TNF), TGF-*β*1 (TGFB1), and CD8+ T cells. We have observed stronger association of CD8+ T cells, IFNG, and TNF with IDO1 compared to TGFB1, in both cohorts (Figure 7). Further, we assessed the correlation between the most significantly enriched metalloproteinases (MMP9, MMP25, ADAMDEC1, ADAMTS2) and IDO1/TNF. Both IDO1 and TNF were strongly associated with metalloproteinase expression (Figure 8). Moreover, we identified IDO1 to be associated with poor prognosis in the training cohort (Supplementary Figure 4).

**Figure 9:**
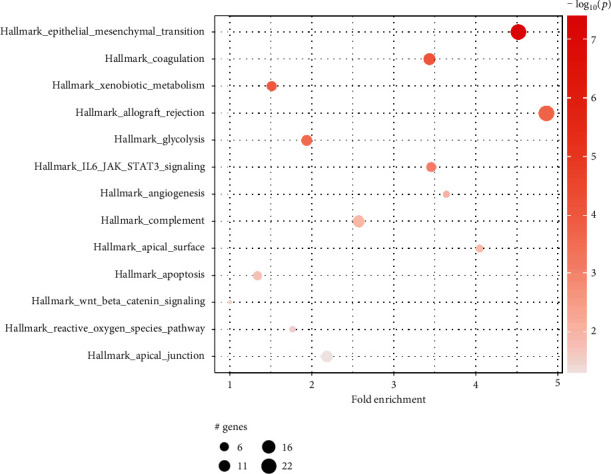
Gene-set enrichment analysis performed using pathfindR and MSigDB Hallmark protein-protein interaction network. Only significantly enriched pathways (*p* < 0.05) are displayed.

We also performed pathway enrichment analysis with pathfindR utilizing MSigDB Hallmark protein-protein interaction network. Interestingly, among the most significantly enriched pathways were epithelial-mesenchymal transition (EMT), glycolysis, and angiogenesis, all of which are associated with increased metastatic potential and worse prognosis (Figure 9). Gene-sets associated with reactive oxygen species (ROS), xenobiotic metabolism, and allograft rejection were also enriched in the high-risk group.

## 4. Discussion

Despite all the recent advances in cancer research, including immune checkpoint targeting and chimeric antigen receptors (CARs), metastatic UM continues to elude the therapy [20, 23, 56, 57]. Curative treatments are scarce and can be rarely achieved, urging for the development of novel therapies. Failed attempts at translating therapies that were successfully used in cutaneous melanoma underline the importance of a comprehensive understanding of UM biology in designing new treatment approaches. Another utterly important task is identification of high-risk patients to allow for cost-effective intensified screening and early metastasis detection.

Immune cells infiltrating the tumor microenvironment have emerged as an important factor determining the cancer patient's prognosis and response to specific therapies, also in uveal melanoma [8, 27–29, 58, 59]. Therefore, understanding the TME architecture and employing gained insights into drug design have become a priority in modern cancer research [24, 60]. The favorable prognostic role of CD8+ T cells has been identified in a number of cancers due to their potent antitumor activity [6, 8, 9]. However, immunosuppressive factors present in the TME can inhibit T cell-mediated response and rewire their activity for the tumor's benefit [11, 12]. In UM, T cell infiltration is associated with poor prognosis [1, 28, 29, 61]. Here, we show that the predominant subset of UM's tumor-infiltrating lymphocytes (TIL), CD8+ T cells, are indeed a negative prognostic factor, as shown in both univariate analysis and in the created 3-cell type prognostic model. Moreover, we lay out the molecular mechanisms that explain the unusual role for CD8+ T cells and propose targets for future preclinical studies in UM.

To gain insights into UM's immunobiology, we investigated the effects of different tumor-infiltrating immune cell subsets on prognosis through a comprehensive analysis of TCGA and GEO databases. Using CIBERSORTx, we estimated the abundance of immune cell subsets in the TME from bulk RNA-seq and microarray data. Then, through a primal-dual active set-based algorithm implemented in BeSS in the training cohort, we have built a prognostic model based on CD8+ T cell, M0 macrophage, and neutrophil abundance scores. T cells and macrophages were associated with a worse prognosis. On the contrary, neutrophils were associated with a favorable outcome. The prognostic value of the created model was assessed using Kaplan-Meier, Cox regression, and ROC analysis. By applying our model to the testing cohort, we have successfully validated its prognostic value through the same evaluation scheme. Significantly, a recently published study on 642 UM patients provided evidence for the superior performance of TCGA-based UM classification compared to standard American Joint Committee on Cancer (AJCC) criteria [33, 62]. While our results need further verification in different experimental settings, the CIBERSORTx algorithm we employed has been extensively validated with techniques such as flow cytometry, mass cytometry, immunohistochemistry, and single-cell RNA-seq by multiple different groups, providing robust data confirming its accuracy [40, 63–65]. Nevertheless, others have already created mRNA/miRNA/DNA-based scores with strong prognostic values in UM tumors [33, 66–72]. Rather than compete with them, our foremost motive for creating the cell type-based prognostic score was to select the cell types playing a prime role in the biology of UM progression to investigate the molecular mechanisms standing behind their prognostic effects and to identify potential future therapeutic targets.

Previous research has identified extensive lymphocyte infiltration as a negative prognostic factor in UM; however, the prognostic role of individual subsets was not assessed [1, 28, 29, 61]. The most predominant lymphocyte population infiltrating the UM TME are CD8+ T cells, while Treg infiltration is observed in 11% of UM samples T [27, 28, 61, 73–76]. It has been shown that despite the presence of clonally expanded T cells capable of recognizing UM neoantigens, they cannot mediate an effective antitumor response [73].

Another dominant subset of tumor-infiltrating immune cells is macrophages. Their infiltration is associated with a worse prognosis, higher microvascular density, and the presence of epithelioid cells [1, 76–78]. Similar to primary tumors, T cells and macrophages are also predominant infiltrating cell types in UM liver metastases [79, 80].

One of the factors associated with an increased immune infiltrate is chromosome 3 monosomy, widely recognized as a negative prognostic factor [76]. The mechanism of observed neutrophil's positive prognostic role is unclear; however, their role in tumor immunity has recently been challenged with an increasing number of papers reporting their antitumor potential [81, 82].

Phase II clinical trial of IL-2-expanded TILs in UM treatment reported partial response in 6 out of 20 patients (30%) and a complete response in one (5%), demonstrating that a subset of patients can potentially benefit from immune-based therapy [83]. Notably, TIL growth from UM samples had a significantly lower success rate compared to CM, indicating the presence of a robust UM-mediated suppression that could not be reverted with cytokine stimulation, partially explaining the overall low treatment efficacy with IL-2 expanded TILs [84].

To delineate the molecular mechanisms shaping the hostile TME in UM and gain insights into the unusual role of immune cells incorporated in the model, we performed differential gene expression and pathway enrichment analysis. We have found that genes associated with suppressing the immune response were enriched in the high-risk group, possibly explaining the lack of positive prognostic effect of CD8+ T cells due to inhibition of their antitumor activity.

The enzyme indoleamine 2,3-dioxygenase (IDO) catalyzes tryptophan degradation, an essential amino acid required for lymphocyte activation and proliferation, while producing highly suppressive kynurenine, resulting in suppression of NK and CD8+ T cell antitumor function [13, 85]. Notably, the IDO expression can be induced by an immunosuppressive cytokine TGF-*β* as well as proinflammatory IFN-*γ* [86]. TGF-*β* is physiologically enriched in the aqueous humor and contributes to sustaining the local immune privilege, explaining the omnipresence of TGF-*β* in UM [87]. Through MHC I downregulation, TGF-*β* was shown to increase UM susceptibility to NK cells, concomitantly increasing their resistance to CD8+ T cells [88]. Low MHC class I levels correlate with improved prognosis in UM patients but also with the scarce lymphocytic infiltrate [75, 89]. Since NK cells are the key effectors in limiting the hematogenous spread of tumor cells, their antimetastatic activity may contribute to positive prognostic effect of the low MHC expression in UM [10, 90].

Triozzi et al. compared the gene expression profile between high- and low-TIL groups in 57 uveal melanoma samples. They found increased expression of IDO1, IFNG, PDL1, CTLA4, and LAG3 in the TIL-rich group and speculated that these changes might be due to the TGF-*β*-Treg axis [91]. Hereby, we show that IFNG, TNF, and CD8+ T cells are more closely correlated with IDO1 than TGFB1. By interpreting our data in the context of available studies on regulation of tryptophan metabolism in UM, we suggest that the primary mechanism is more likely to IFN-*γ*- and TNF-*α*-dependent, both of which are produced by tumor-infiltrating cytotoxic lymphocytes. This is supported by in vitro studies on UM cell lines, which have identified both TNF-*α* and IFN-*γ* as potent inducers of the IDO1 expression, synergistic in their action [92]. Another group has also reported a strong correlation between IDO1 and IFNG mRNA in UM [93]. In UM, the IDO1 expression is not constitutive and requires prior stimulation, explaining the low expression in the low-risk CD8+ T cell-depleted group [85]. IDO1 expression correlates with the expression of checkpoint molecules, CTLA-4 and PD-L1 [93]. Significantly, metastatic UM samples are characterized by the high IDO1 expression, implicating that altering the tryptophan metabolism is an intrinsic immunoevasion mechanism characteristic for advanced UM [94].

The prognostic role of IDO1 is not fully understood. Liang et al. conducted an extensive bioinformatic analysis of GSE22138 and GSE27831 to evaluate the prognostic role of the IDO1 expression, and no simple associations were discovered [93]. Notably, the authors used the occurrence of metastasis as a primary endpoint, while no association with OS was investigated. We show that the IDO1 expression is strongly associated with poor overall survival, prompting further investigations of IDO's possible prognostic role. IDO1 and CD8+ T cell signature prognostic roles were also identified by other groups in the analysis of the same datasets [70, 71]. Moreover, IDO1 was among the most highly expressed genes in UM with chromosome 3 monosomy, a group characterized by an increased metastatic risk [33].

We have also identified kynureninase (KYNU), an enzyme involved in kynurenine degradation, as a novel player in UM TME. We found expression of KYNU to be downregulated in the high-risk group, and we speculate that its decreased activity allows maintaining higher kynurenine concentration, thus more profound immunosuppression. Moreover, 3-hydroxyanthranilic acid (3-HAA) produced by KYNU through kynurenine degradation was shown to increase proinflammatory cytokine synthesis in keratinocytes, endothelial cells, and decrease Foxp3 expression in T cells, augmenting the inflammatory response [95]. Thus, a decrease in KYNU increases the concentration of suppressive kynurenine and decreases proinflammatory 3-HAA. Interestingly, KYNU/IDO mRNA ratio was found to be specific for different groups of diseases. Inflammatory and infectious diseases are characterized by a higher ratio (above 1) than neoplastic diseases [95]. Altogether, we show that UM can rewire the tryptophan metabolism at multiple levels, suppressing the immune response.

Metalloproteinases are again emerging as targets in cancer therapy [96]. Their role goes beyond being simple effectors of angiogenesis and metastasis as they have been proposed to play a role in regulating immune response [97–100]. We have identified MMP-1, MMP-9, MMP-12, MMP-25, ADAMTS2, ADAMDEC1, and others to be enriched in the high-risk subset of UM patients. The prognostic role of MMP-2, MMP-9, and MMP-14 in UM was recognized in several studies due to their crucial role in promoting cancer cell motility and angiogenesis, ultimately leading to the development of metastases [101–108]. Furthermore, in an MMP-2-dependent manner, TGF-*β* was shown to alter the adhesome of UM cells, increasing their adhesive properties to hepatic endothelium, implicating an essential role in the development of UM liver metastases [109]. It is worth noting that the development of metastasis is the most critical event in the UM course, determining the patient's prognosis [1, 3]. Moreover, ADAMTS-2, together with other ADAMTS protease family members, was recently associated with UM stemness, endothelial-like phenotype, increased angiogenesis, and poor prognosis [110–112]. Therefore, metalloproteinase pharmacological inhibition was recognized as a promising therapeutic intervention in in vitro UM models [113, 114]. We show that the high-risk group is associated with a substantially enhanced metalloproteinase profile, which likely plays a dominant role in driving UM metastases.

Similarly to IDO1, the MMP expression can also be induced by TILs in a TNF-*α*-dependent manner [115]. We show that TNF correlates with the expression of MMP9, MMP25, ADAMDEC1, and ADAMTS2. In addition, IDO1 overexpression alone has been shown to increase the expression of MMP-1, MMP-3, and MMP-9 in in vitro studies, providing a direct link between the two distinct groups of genes [116–119]. Our findings support that by showing the close positive association between IDO1 and metalloproteinase expression. Based upon our findings and the published in vitro data, it is likely that coexpression of both gene groups in the CD8+ T cell-rich group is causatively related.

The genomic landscape of UM has a significant impact on tumor development. Chromosomal aberrations affecting chromosome 3 and 8q regions, in particular, are linked to poor prognosis [5, 24, 33, 120]. The amplification of 8q segment and loss of chromosome 3 were also shown to correlate with increased infiltration of macrophages and T cells [24, 76, 121]. This, in turn, impedes the discrimination between the protumorigenic effects of chromosomal aberrations and tumor-infiltrating immune cells. In our study, 8q amplification and chromosome 3 loss weakly correlate with the created risk score (Supplementary Figure 3). However, when we applied our model on the training cohort split into subgroups based on chromosome 3 and 8q copy-number status, we observed that our model retained its strong prognostic value in the subgroups characterized by high-risk chromosomal aberrations (Supplementary Table 2). This implicates that, at least partially, the infiltration of CD8+ T cells, M2 macrophages, and neutrophils exhibits genomic-independent protumorigenic effects. Nevertheless, it is currently impossible to precisely distinguish the impact of genomic aberrations from the effects of tumor-infiltrating cells on tumor development and prognosis. Despite strong correlation and supporting in vitro data, observed effects of immune cell signatures might still be due to concomitance with other high-risk factors, urging for further studies.

Additionally, because of the well-established immunobiology of cutaneous melanoma, we have assessed how our model and its individual components are related to prognosis. Intriguingly, all the prognostic trends were reversed, with the effects of CD8+ T cells and neutrophils being statistically significant. Parallel tumor-infiltrating lymphocyte (TIL) profiling of UM and CM samples revealed that despite a similar extent of CD8+ T cell infiltration, the expression of PD1-PD-L1 was much lower in UM samples what together with lower mutational burden may explain the inefficacy of checkpoint inhibition as a treatment [20, 21, 84]. We show that despite a resembling cellular origin, from the immunobiological point of view, uveal and cutaneous melanomas are two very distinct tumor types and should be treated as such when designing and testing novel immunotherapies. The immense differences in their mutational burden and TME architecture can serve as hints for planning new clinical trials [20, 25, 84].

## 5. Conclusions

In conclusion, we have identified three cell types crucial for UM prognosis, utilizing an immune cell score based on the negative role of CD8+ T cells and M0 macrophages and the positive role of neutrophils in the UM TME. Then, by studying the molecular mechanisms associated with the prognostic role of included cell types, we identified altered tryptophan metabolism and increased expression of metalloproteinases to be the factors most likely accountable for the hampered antitumor activity of CD8+ T cells as well as their apparent tumor-promoting role in UM. We set out the hypothesis of the central role of CD8+ T cells in TIL-rich UM progression. Through IFN-*γ* and TNF-*α*, CD8+ T cells can trigger an adaptative response in UM, altering the tryptophan metabolism as an immunoevasion mechanism. Besides suppressing the immune response, IDO1 together with T cell-derived TNF-*α* lead to upregulation of metalloproteinases, which in turn increase the metastatic potential of UM. Since the immunological milieu of metastatic UM's was shown to be similar to primary UM's, these findings emphasize that inhibition of IDO1 and metalloproteinase activity might be a beneficial approach to restore T cell-mediated immunosurveillance in TIL-rich UM patients.

## Figures and Tables

**Table 1:** Clinical and pathological characteristics of uveal melanoma patients at the time of diagnosis. Missing data is marked as NA (not available).

Characteristics	Training cohort (TCGA-UVM, *n* = 71)	Testing cohort (GSE22138, *n* = 60)
Age at diagnosis (years)		
Mean (SD)	61 (14)	61 (13)
Min-max	22-86	29-85
Gender		
Female	31 (43.66%)	22 (36.67%)
Male	40 (56.34%)	38 (63.33%)
Clinical stage		
I	0 (0.00%)	NA
II	31 (43.66%)	NA
III	36 (50.71%)	NA
IV	4 (5.63%)	NA
T classification		
T1	0 (0.00%)	NA
T2	3 (4.22%)	NA
T3	32 (45.07%)	NA
T4	34 (47.89%)	NA
Tx	2 (2.82%)	NA
N classification		
N0	67 (94.37%)	NA
N1	0 (0.00%)	NA
Nx	4 (5.63%)	NA
M classification		
M0	64 (90.14%)	NA
M1	3 (4.22%)	NA
Mx	4 (5.63%)	NA
Extrascleral extension		
No	60 (84.51%)	47 (78.33%)
Yes	7 (9.86%)	4 (6.67%)
Unknown	4 (5.63%)	9 (15.00%)
Tumor basal diameter (mm)		
Mean (SD)	17 (4)	15 (4)
Range	8-25	9-23
Tumor thickness (mm)		
Mean (SD)	11 (3)	12 (2)
Range	4-16	6-17

**Table 2:** Selected differentially expressed genes (DEGs) between high- and low-risk groups in the training cohort. The genes were selected based on the biological function.

Gene symbol	Gene name	Fold change (log2)	Adjusted *p* value
*KYNU*	Kynureninase	-1.755	0.00786
*IDO1*	Indoleamine 2,3-dioxygenase 1	2.326	0.01879
*MMP9*	Matrix metalloproteinase 9	2.745	<0.0001
*MMP1*	Matrix metalloproteinase 1	1.761	0.029
*MMP12*	Matrix metalloproteinase 12	4.562	0.00065
*MMP25*	Matrix metalloproteinase 25	1.690	0.00054
*ADAMTS2*	ADAM metallopeptidase with thrombospondin type 1 motif 2	1.696	0.00251
*ADAMDEC1*	ADAM like decysin 1	2.863	0.005
*ADAM11*	ADAM metallopeptidase domain 11	0.975	0.0476
*ADAMTS8*	ADAM metallopeptidase with thrombospondin type 1 motif 8	0.931	0.0207
*ADAMTS4*	ADAM metallopeptidase with thrombospondin type 1 motif 4	0.928	0.0047
*ADAMTS9*	ADAM metallopeptidase with thrombospondin type 1 motif 9	0.962	0.0064
*ADAMTS15*	ADAM metallopeptidase with thrombospondin type 1 motif 15	0.969	0.0056

## Data Availability

The gene expression data used in this study can be found in the The Cancer Genome Atlas repository with cohort name GDC TCGA-UVM/TCGA-PANCAN and in the GEO database with cohort name GSE22138.

## Supplementary Materials

Supplementary Materials Supplementary Figure 1: distribution of immune cell subsets in the training cohort. Supplementary Figure 2: distribution of immune cell subsets between the high- and low-risk groups. Supplementary Figure 3: correlation between the risk score and commonly detected chromosomal aberrations. Supplementary Figure 4: the prognostic effect of IDO1 expression assessed with Kaplan-Meier analysis in the training cohort. Supplementary Table 1: univariate Cox regression analysis of 22 cell types in the training cohort. Supplementary Table 2: assessment of the created model's prognostic value in the training cohort split into subgroups by commonly detected chromosomal aberration status.
