# Supplementary material for: Bioinformatic Analysis Reveals Central Role for Tumor-Infiltrating Immune Cells in Uveal Melanoma Progression
Source: J Immunol Res. 2021 Jun 11;2021:9920234. doi: 10.1155/2021/9920234 (PMC8214507; doi:10.1155/2021/9920234)
Supplement: Supplementary Materials — Supplementary Figure 1: distribution of immune cell subsets in the training cohort. Supplementary Figure 2: distribution of immune cell subsets between the high- and low-risk groups. Supplementary Figure 3: correlation between the risk score and commonly detected chromosomal aberrations. Supplementary Figure 4: the prognostic effect of IDO1 expression assessed with Kaplan-Meier analysis in the training cohort. Supplementary Table 1: univariate Cox regression analysis of 22 cell types in the training cohort. Supplementary Table 2: assessment of the created model's prognostic value in the training cohort split into subgroups by commonly detected chromosomal aberration status. [file 9920234.f1.docx]

| **Cell type** |  | **Univariate Cox regression** | |
| --- | --- | --- | --- |
|  |  | **p-value** | **Hazard Ratio (95% CI)** |
| T cells CD8 |  | 0.0011 | 6.24 (2.08-18.75) |
| Macrophages M0 |  | 0.0014 | 5.03 (1.86-13.58) |
| T cells regulatory Tregs |  | 0.0085 | 3.62 (1.39-9.46) |
| Mast cells activated |  | 0.0105 | 3.4 (1.33-8.67) |
| Macrophages M1 |  | 0.0863 | 2.19 (0.89-5.37) |
| T cells follicular helper |  | 0.1097 | 2.1 (0.85-5.22) |
| T cells gamma delta |  | 0.1420 | 1.96 (0.8-4.81) |
| Macrophages M2 |  | 0.3259 | 1.55 (0.65-3.69) |
| Plasma cells |  | 0.982 | 1.01 (0.43-2.39) |
| NK cells activated |  | 0.9544 | 0.98 (0.41-2.31) |
| T cells CD4 memory activated |  | 0.9381 | 0.97 (0.41-2.3) |
| B cells memory |  | 0.9049 | 0.95 (0.4-2.24) |
| Monocytes |  | 0.4512 | 0.72 (0.3-1.71) |
| NK cells resting |  | 0.2422 | 0.59 (0.24-1.43) |
| Dendritic cells resting |  | 0.2026 | 0.56 (0.23-1.36) |
| B cells naive |  | 0.1503 | 0.51 (0.21-1.27) |
| T cells CD4 naive |  | 0.3386 | 0.37 (0.05-2.8) |
| T cells CD4 memory resting |  | 0.0037 | 0.2 (0.07-0.59) |
| Neutrophils |  | 0,0008 | 0.17 (0.06-0.48) |
| Eosinophils |  | 0.0031 | 0.16 (0.05-0.54) |
| Dendritic cells activated |  | 0.0006 | 0.12 (0.03-0.4) |
| Mast cells resting |  | 0.0003 | 0.11 (0.03-0.36) |

Supplementary Table 1: Univariate Cox regression analysis of 22 cell types in the training cohort (overall survival). Patients were divided into two groups based on the median abundance estimate of each cell type.

| **Group** |  | **p-value** | **Hazard Ratio (95% CI)** |
| --- | --- | --- | --- |
| Training cohort (n = 71) |  | 0.0007 | 9.67 (2.603 - 35.90) |
| Training cohort 8q CN <3 (n = 18) |  | 0.999 | 6.725e+08 (0 – Inf) |
| Training cohort 8q CN >=3 (n = 53) |  | 0.00051 | 10.1874 (2.751- 37.72) |
| Training cohort 3 CN <2 (n = 32) |  | 0.00867 | 5.7983 (1.561 - 21.54) |
| Training cohort 3 CN >=2 (n = 33) |  | 0.999 | 7.453e+08 (0 – Inf) |

Supplementary Table 2: Cox regression analysis of the created model in the training cohort (overall survival) divided by 8q segment copy-number (CN) status (cutoff CN >=3) and chromosome 3 CN status (cutoff CN <2).


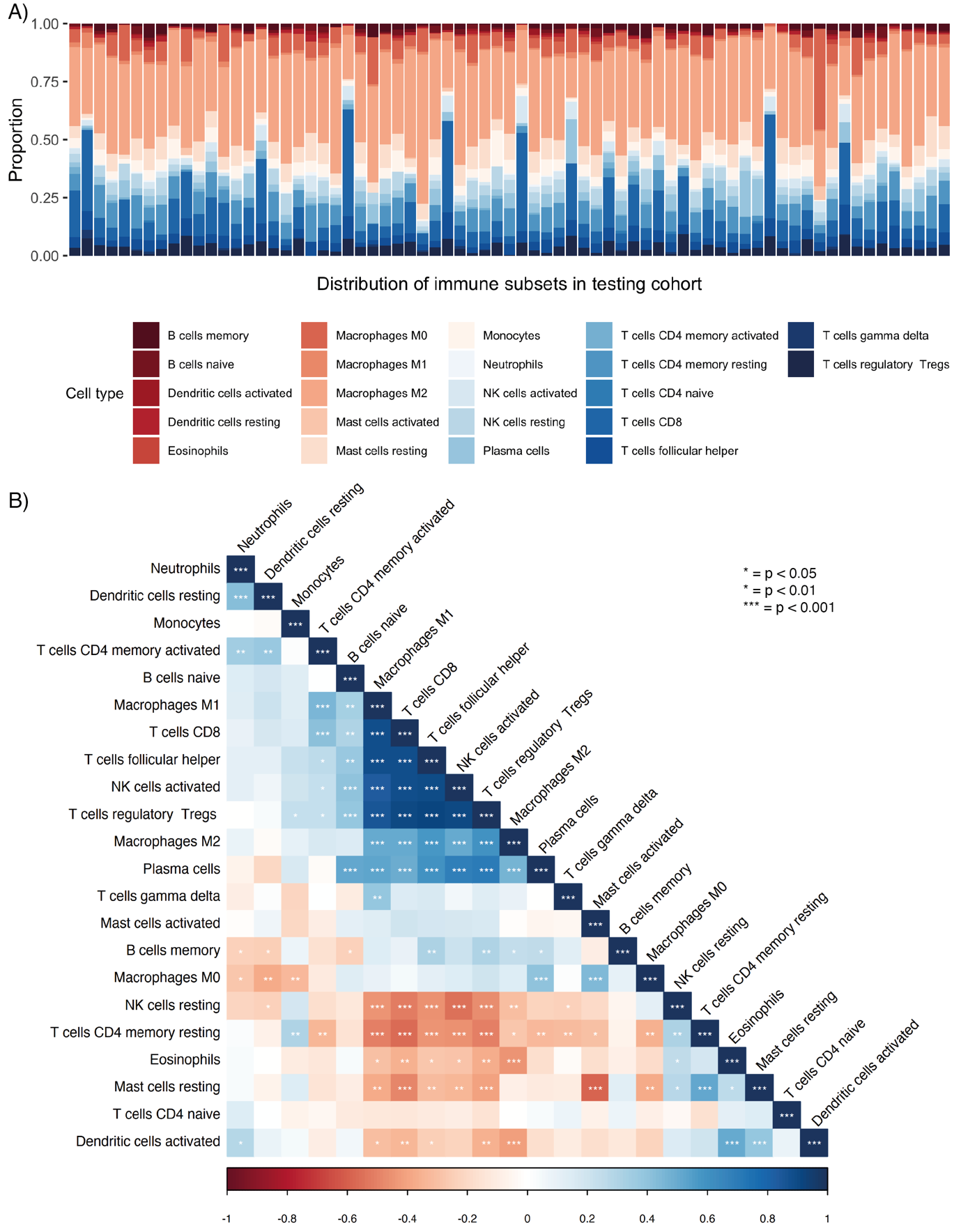
Supplementary Figure 1: Distribution of immune cell subsets (A) and correlation between the cell subsets (B) in the training cohort.

**
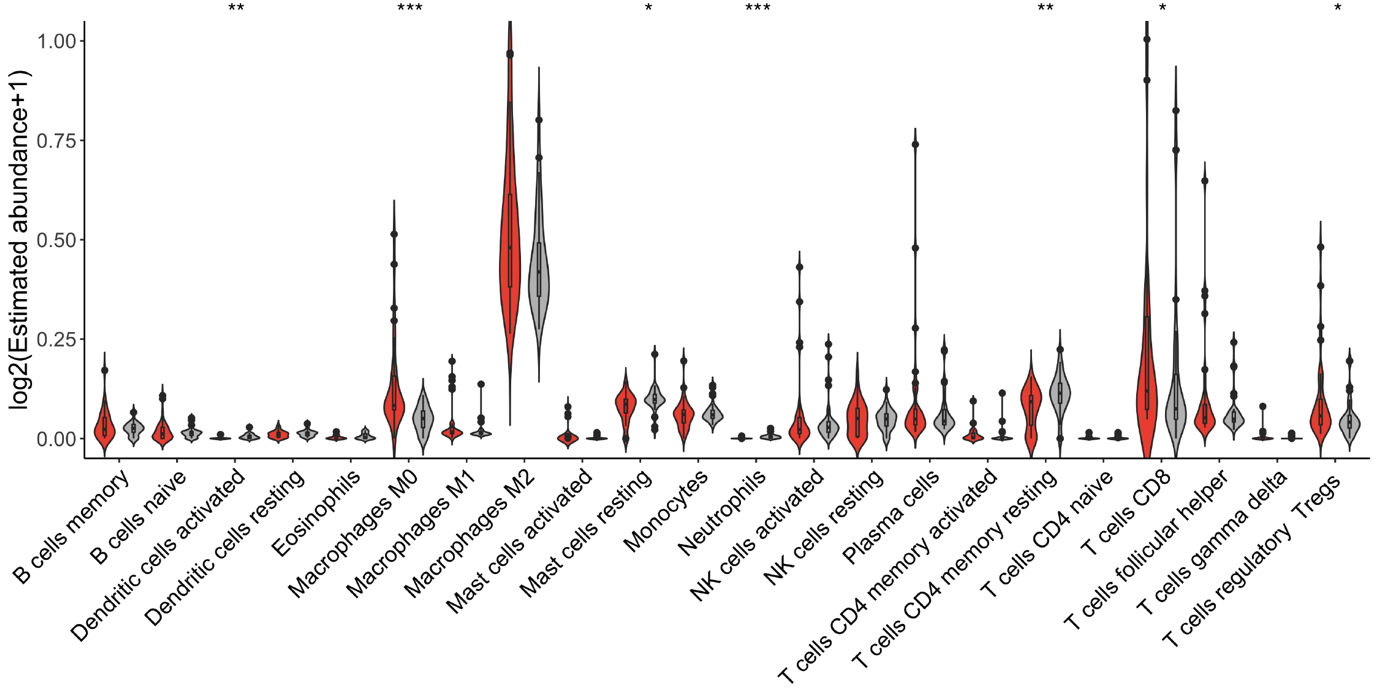
**Supplementary Figure 2: Distribution of immune cell subsets between the high- (red) and low-risk (gray) groups in the training cohort. Statistically significant differences were marked with * (p < 0.05), ** (p < 0.01), or *** (p < 0.001).

**
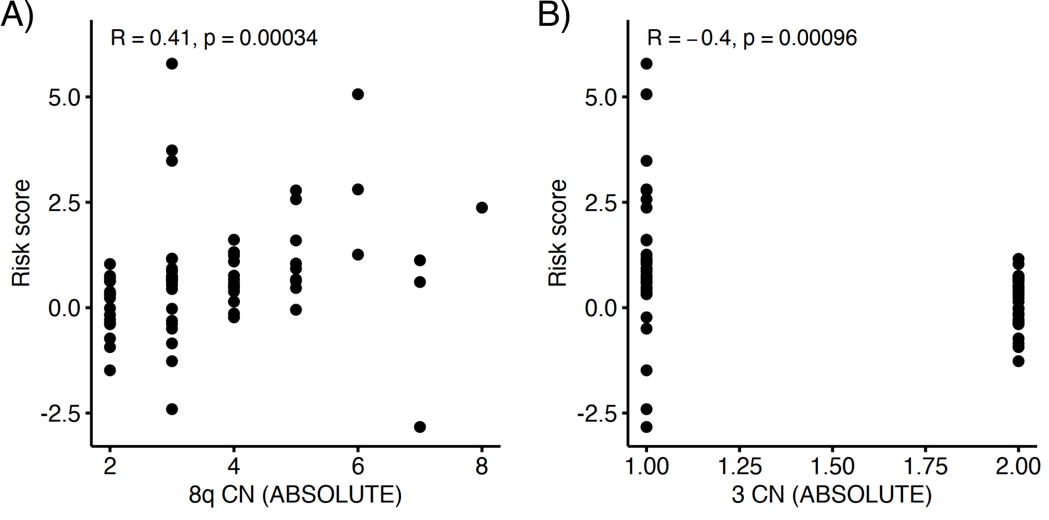
**

Supplementary Figure 3: Correlation between the created risk score and a) 8q segment b) chromosome 3 copy-number (CN) in the training cohort.

**
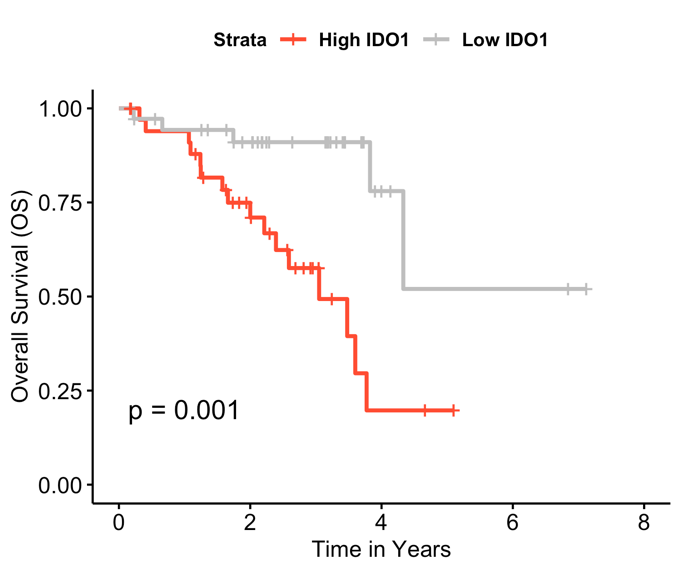
**

Supplementary Figure 4: The prognostic effect of IDO1 expression assessed with Kaplan-Meier analysis in the training cohort. Differences between the groups were detected by the log-rank test.
